# A comparative study of femtosecond pulsed laser ablation of meloxicam in distilled water and in air

**DOI:** 10.1038/s41598-023-36922-7

**Published:** 2023-06-23

**Authors:** Eszter Nagy, Judit Kopniczky, Tamás Smausz, Máté Náfrádi, Tünde Alapi, János Bohus, Viktor Pajer, Piroska Szabó-Révész, Rita Ambrus, Béla Hopp

**Affiliations:** 1 Department of Optics and Quantum Electronics, University of Szeged, Dóm tér 9, Szeged, 6720 Hungary; 2 Department of Inorganic and Analytical Chemistry, University of Szeged, Dóm tér 7, Szeged, 6720 Hungary; 3 ELI ALPS, ELI-HU Non-Profit Ltd., Wolfgang Sandner utca 3, Szeged, 6728 Hungary; 4 Institute of Pharmaceutical Technology and Regulatory Affairs, University of Szeged, Eötvös utca 6, Szeged, 6720 Hungary

**Keywords:** Nanomedicine, Drug delivery, Biomedical engineering

## Abstract

The increasing prevalence of water insoluble or poorly soluble drugs calls for the development of new formulation methods. Common approaches include the reduction of particle size and degree of crystallinity. Pulsed laser ablation is a clean technique for producing sub-micrometre sized drug particles and has the potential to induce amorphization. We studied the effect of femtosecond pulsed laser ablation (ELI ALPS THz pump laser system: λ_c_ = 781 nm, τ = 135 fs) on meloxicam in distilled water and in air. The ablated particles were characterized chemically, morphologically and in terms of crystallinity. We demonstrated that femtosecond laser ablation can induce partial amorphization of the particles in addition to a reduction in particle size. In the case of femtosecond pulsed laser ablation in air, the formation of pure meloxicam spheres showed that this technique can produce amorphous meloxicam without the use of excipients, which is a unique result. We also aimed to describe the ablation processes in both investigated media.

## Introduction

The significant increase in the number of water insoluble or poorly soluble active pharmaceutical agents (APIs) has led to the development of new techniques aiming to improve the solubility and bioavailability of these substances^1–3^ Common approaches include, for example, the use of inclusion complexes with cyclodextrin^3,4^, salt-formation^5^ or the application of co-solvents^6^. Further possibilities are increasing the active surface area by reducing the particle size^7,8^ and concepts that disrupt the crystalline order^9^. Particle size reduction^10^ can be achieved by milling^11,12^, high-pressure homogenization^13^, spray drying^14,15^ or freeze-drying^16–18^, liquid anti-solvent crystallization^19,20^ or supercritical fluid-based techniques^21,22^, applied separately or combined^23^. In addition to direct amorphization processes (for example polymeric amorphous solid dispersions^9,24^ and hot melt technologies^25–27^), particle size reduction methods may also influence the crystallinity of the samples.

Laser ablation is a well-established material processing technique with great potential and growing interest in life sciences^28,29^. Pulsed laser ablation can be appealing for pharmaceutical developments as it is a green, clean, straightforward and fast technique with easily adjustable parameters (e.g. laser parameters, medium). Ultrashort laser pulses of femtosecond lasers can be applied as a novel top-down method to obtain submicrometer-sized drug particles and compared to nanosecond pulses the ablation mechanism can be significantly different due to the six orders of magnitude shorter pulse length^30–33^.

Femtosecond laser ablation has been shown to be effective in reducing the size of drug particles dispersed in liquid^34–38^. However, often significant degradation of the API occurred, which limits the use of this technique. Therefore, we decided to study the pulsed laser ablation of a bulk (compressed API) target, which has been less studied^39^.

As model drug, we choose meloxicam, a commonly used nonsteroidal anti-inflammatory drug (NSAID) used to treat pain and inflammation in rheumatic diseases and osteoarthritis. However, due to its low dissolution and low solubility it takes too long to reach its peak plasma concentration for a quick onset effect. Therefore, there is a great need to develop meloxicam formulations with high rates of intestinal absorption and retention and enable a quick onset of the therapeutic effect^40^.

Laser ablation may provide a novel method to generate a pharmaceutical intermediate product with improved physico-chemical properties, which could then be applied to the preparation of new dosage forms. Since meloxicam has a melting temperature (T_m_) of 254 °C, which is also considered to be its degradation temperature^41^, the careful selection of laser parameters when using laser ablation for particle size reduction is crucial.

Our present work focuses on the femtosecond pulsed laser ablation of pressed meloxicam pastilles in distilled water (pulsed laser ablation in liquid, PLAL) and in air (pulsed laser ablation, PLA). This study is an extension of our previous investigation^42^, and it complements our studies on nanosecond pulsed laser ablation of meloxicam in liquid^43,44^, in air^45^ and ibuprofen in vacuum^46^. In the case of femtosecond pulsed laser ablation, the investigations indicated partial amorphization of meloxicam^42^. Since crystallinity plays a key role in the solubility and bioavailability of drug substances, and amorphous meloxicam could previously only be prepared by the addition of excipients^47,48^, we decided to try to enhance amorphization while using only pure meloxicam. We increased the repetition rate of the laser pulses by three orders of magnitude (to 1 kHz) and investigated how it affected the amorphization of meloxicam. We inspected the chemical composition, the morphology, size, and crystallinity of the generated particles to see whether they could be suitable for future pharmaceutical applications. The experimental results are interpreted in the light of the different particle formation processes in air and in liquid.

## Materials and methods

### Meloxicam (mx.)

As a model compound, we used a poorly water soluble drug, meloxicam (C_14_H_13_N_3_O_4_S_2_; 4-hydroxy-2-methyl-N-(5-methyl-2-thiazolyl)2H-benzothiazine-3-car-boxamide-1,1-dioxide). Meloxicam purchased from Sigma-Aldrich Chemistry (Darmstadt, Germany) was a yellow, 100% crystalline powder with a pharmaceutical grade above 99% and particle sizes in the micrometre range: d(0.1) = 9.5 µm, d(0.5) = 27.5 µm and d(0.9) = 58.7 µm.

### Target preparation for pulsed laser ablation

Our targets were pastilles pressed from 300 mg meloxicam powder using 15 kN compacting force with a KORSCH EK-0 Tablet Press machine (KORSCH AG—Berlin, Germany).

### Laser source

The ELI ALPS THz pump laser system (Amplitude Technologies, Lisses, France) is a diode laser pumped (Terra 527-50-M provided by Continuum) Ti:sapphire-based laser, seeded by a mode-locked laser oscillator (C-Fiber 780 High Power Femtosecond Laser provided by Menlo Systems GmbH, Planegg, Germany). The central wavelength is 781 nm with 13.3 nm bandwidth. The transform limited pulses are 92.8 fs. We conducted the experiments with negatively chirped 135 fs pulses. The pulse energies were around 4 mJ (> 4 mJ) with 1 kHz repetition rate.

### Pulsed laser ablation setup

**Figure 1:**
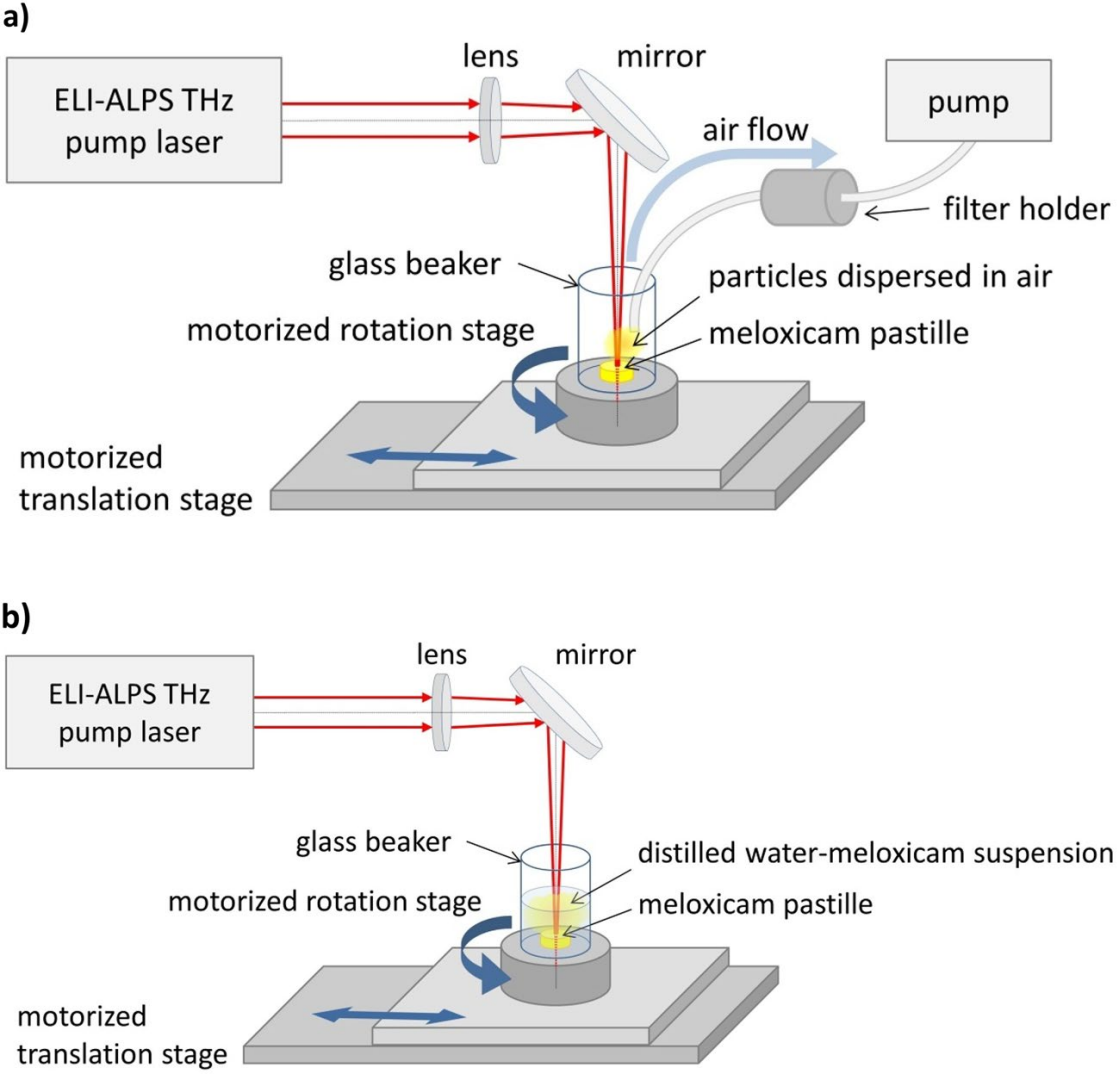
Experimental setup for (**a**) pulsed laser ablation in air (PLA) and (**b**) in distilled water (PLAL).

We used the same setup for pulsed laser ablation in air (PLA) (Fig. 1a) and in liquid (PLAL) (Fig. 1b), but the medium and the particle collection method were different.

The laser beam was guided by a mirror (UM15-45A, Thorlabs Inc., Newton, NJ, USA) and focused slightly under the surface of the target by a plano-convex lens with a focal length of 200 mm (LA1979-B-N-BK7, Thorlabs Inc., Newton, NJ, USA). The average fluence at the target was around 1.9 J/cm^2^ throughout the experiments.

To enable scanning of the pastille surface, the target was fixed to the bottom of a glass beaker placed on a combination of a motorized rotation and translation stage (LTM 120-xxx-HSM-V15SubDF-V6).

In the case of PLA, a constant air flow steered the generated particles to a membrane filter (pore size: 1.2 µm) (MCE membrane filter, 420.MF025ME120 Labsystem Kft.). As particles accumulated on the filter, the pores eventually clogged up, and particles of all sizes were collected for further investigations. Each sample was generated by approximately 10,000 pulses.

In the case of PLAL, the meloxicam pastille was covered with distilled water, forming a 5 mm thick liquid layer over the target. The ejected particles accumulated in the water, which was changed after about 300,000 pulses. (Each particle suspension was produced by approximately 600,000 pulses.) The PLAL generated particles for FTIR, Raman, XRPD and DSC analysis were extracted from the suspension by evaporating the water content at 50 °C in a laboratory oven (CARBOLITE 2416, Thermal Engineering Services Ltd., Worcester, England) within approximately 12 h.

### Fourier transform infrared spectroscopy (FTIR)

A few mg of the particles produced by PLA and collected on the membrane filter were scraped off and mixed with 150 mg KBr. The mixture was ground in an achate mortar and pressed into a disk with 10 kN force.

The PLAL generated dried particles were mixed with KBr and pressed into disks for FTIR analysis the same way as written above.

FTIR spectra were recorded with an FTIR spectrometer (Thermo Nicolet AVATAR 330, LabX Midland, ON, Canada) in the 4000–400 cm^−1^ wavenumber range, at a resolution of 4 cm^−1^, averaging 128 scans per measurement, with baseline correction.

### Raman spectroscopy

For Raman spectroscopy, the PLA generated particles were collected from the surface of the clogged membrane filter, while the PLAL generated particles were extracted from the suspension via evaporation.

Raman spectra were recorded with a Thermo Scientific™ DXR™ Raman microscope (Thermo Fisher Scientific Inc., Waltham, MA, USA). For excitation, λ = 780 nm laser radiation with 2 mW laser power and an estimated spot diameter of 3.1 µm was used. Raman spectra in the range of 2500–500 cm^−1^ were investigated using a 400 lines per mm grating with a resolution of 4.7–8.7 cm^−1^. Each spectrum was acquired by 20 scans with a 2 s integration time.

### High performance liquid chromatography and mass spectrometry (HPLC–MS) studies

To separate and identify the ablation products, HPLC–MS was applied. The PLA/PLAL generated particles were dissolved in the mixture of 10 ml distilled water and 10 ml acetonitrile. Before the HPLC analysis, the remaining solid particles were removed by filtration through a syringe filter (mean pore diameter: 0.22 µm).

The measurements were carried out using an Agilent 1100 HPLC system equipped with a diode array detector (DAD) and a 1956A Agilent LC/MSD/VL mass spectrometer (MS) (Agilent Technologies, Palo Alto, CA, USA). The column was a Kinetex 2,6u XB-C18 100A (Phenomenex), thermostated at 35 °C. The eluent consisted of 40 v/v% methanol and 60 v/v% water with formic acid (0.1 v/v %), applying a flow rate of 0.80 ml min^−1^. Detection was performed at 210 nm, 270 nm, and 350 nm. For the MS measurements the following parameters were applied: electrospray ionization (ESI) in positive ion mode, 300 °C nitrogen drying gas, 3500 V capillary voltage and 50 V fragmentor voltage.

### Scanning electron microscopy (SEM)

We investigated morphology and particle size with scanning electron microscopy (SEM). A small piece of the particle covered filter provided a sample of the PLA generated particles. On the other hand, the PLAL generated particles were obtained for SEM by drying droplets of the colloidal suspension on a silicon plate immediately after ablation. The solid residue obtained after evaporating the water content of the suspension in a 50 °C oven was also investigated.

Before imaging, all samples were gold coated with a sputter-coater (Bio-Rad SC 502, VG Microtech, Uckfield, UK). The SEM studies were performed with a Hitachi S-4700 SEM system (Hitachi S4700, Hitachi Scientific Ltd., Tokyo, Japan).

### X-ray powder diffraction (XRPD)

We described the crystallinity with an X-ray powder diffractometer. The samples for XRPD were identical to the ones for Raman spectroscopy. For comparability, an equal amount of solid was measured from the PLA and PLA generated, as well as from the reference meloxicam powder. Samples were characterized with a BRUKER D8 Advance X-ray powder diffractometer (Bruker AXS GmbH, Karlsruhe, Germany) with Cu Kα radiation (λ = 1.5406 Å) and a VÅNTEC-1 detector, by scanning the samples at 40 kV and 40 mA, with a 2θ angular range of 3° to 40°, at a scan speed of 0.1 s per step and a step size of 0.0074°.

### Differential scanning calorimetry (DSC)

The samples for the DSC investigations were identical with the ones used for Raman spectroscopy. We studied the temperature dependent changes of the laser ablation generated samples with a differential scanning calorimeter (Mettler Toledo DSC 821e, Mettler Inc., Schwerzenbach, Switzerland). During the measurements, an empty pan was used as reference, and as sample, about 2–4 mg of powder (original meloxicam powder, PLA or PLAL generated particles) was weighed into a DSC sample pan, which was hermetically sealed, and the lid pierced. The samples were examined in an inert atmosphere under constant argon purge, in the 25–300 °C temperature range at a heating rate of 10 °C per min.

## Results

### Chemical composition (FTIR, Raman, HPLC–MS)

FTIR, Raman spectroscopy and HPLC–MS studies were performed to characterize the chemical composition of the particles generated by laser ablation.

**Figure 2:**
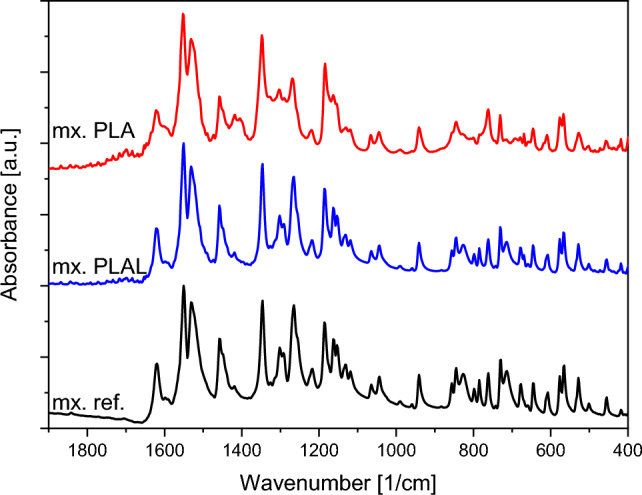
FTIR spectra of PLA (mx. PLA) and PLAL (mx. PLAL) generated particles and the original meloxicam (mx. ref.) powder as reference. The FTIR spectra were normalized in all cases to the peak at around 1550 cm^−1^.

The FTIR spectra of the PLA and PLAL generated particles were compared with the meloxicam reference (Fig. 2).

The FTIR spectra of the PLAL generated particles matched the spectra of the original meloxicam powder very well: the characteristic peaks were at the expected wavenumbers and the relative peak intensities were the same.

The spectra of the PLA generated particles showed close resemblance to the spectra of the meloxicam reference regarding the presence of the peaks, but the relative size of the peaks varied slightly. The signal of the PLA sample increased at around 1600 cm^−1^, 1400 cm^−1^, 1320 cm^−1^, 1280 cm^−1^, 1170 cm^−1^, 850 cm^−1^, 830 cm^−1^, and decreased at 715 cm^−1^, compared to the reference.

**Figure 3:**
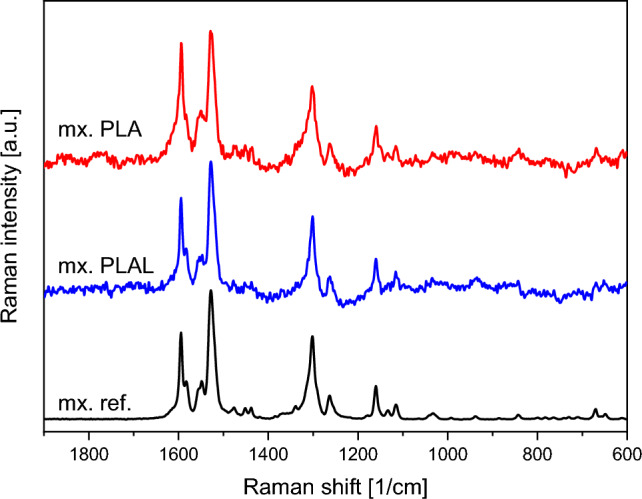
Raman spectra of PLA (mx. PLA) and PLAL (mx. PLAL) generated particles and the original meloxicam (mx. ref.) powder as reference. The Raman spectra were normalized to the peak at around 1530 cm^−1^.

The Raman spectra of neither the PLA, nor the PLAL generated particles showed significant deviations from the original meloxicam spectrum: the Raman shift and relative intensities of the characteristic peaks corresponded well to the reference (Fig. 3). Only minor intensity changes were present (e.g. at 1600 cm^−1^).

Based on HPLC–MS analyses, meloxicam and meloxicam impurity B were identified in the laser ablated samples. Meloxicam impurity B (chemical name: 2-Amino-5-methylthiazole) is a subsection of the meloxicam molecule, one of the major alkaline metabolites of meloxicam, a heterocyclic building block, a pharmaceutical primary standard^49–51^. No other degradation products^51,52^ were detected.

**Figure 4:**
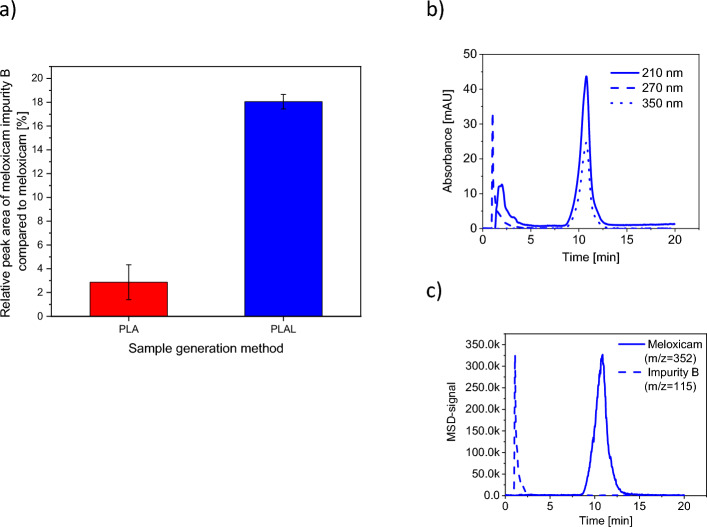
HPLS-MS analysis. (**a**) The relative peak areas of meloxicam impurity B compared to meloxicam [(Area_impurity_/Area_meloxicam_) × 100%] in case of PLA and PLAL generated particles. (**b**) The chromatogram (DAD signal), and (c) the MSD signal detected in SIM mode for the PLAL generated particles.

The amount of meloxicam impurity B was given relative to the peak area of meloxicam detected in SIM mode (Fig. 4), considering the peak area of meloxicam as 100%. The error bars correspond to the standard deviation of data obtained from measurements of three samples prepared independently, using the same process. In the case of PLA, the relative peak area of meloxicam impurity B was less than 5% of the meloxicam peak area, in accordance with the degree of degradation observed in case of nanosecond pulsed laser ablation in distilled water^44^. In case of PLAL, the relative peak area of meloxicam impurity B was ∼ 20% of the meloxicam peak area. However, the FTIR and Raman spectra do not show this compositional difference between the PLA and PLAL samples, indicating the limitation of these techniques. But it should also be noted that since meloxicam impurity B forms part of the meloxicam molecule, only subtle changes could be expected in the FTIR and Raman spectra. A further argument that degradation is not the cause of the minor differences observed in the FTIR and Raman spectra of the samples compared to the reference is that they were more pronounced for PLA, although HPLC–MS measurements showed that PLAL generated more degradation products.

### Morphology, particle size and process of particle generation (SEM, XRPD, DSC)

**Figure 5:**
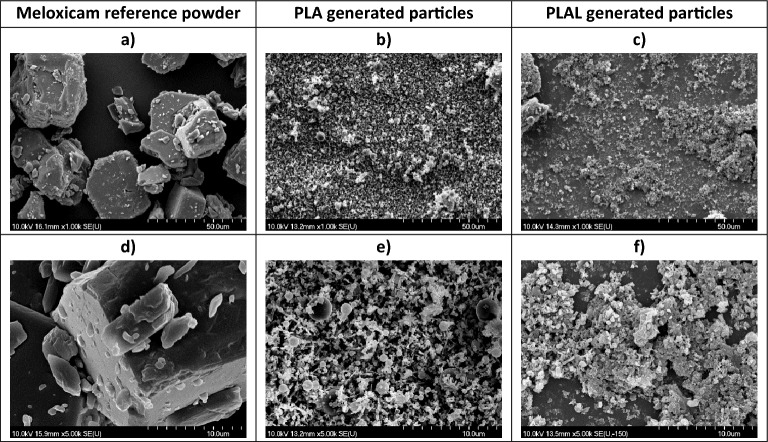
SEM images of the commercially available meloxicam powder (mx. ref.) and the particles generated by PLA and PLAL. Magnification: (**a**)–(**c**): 1 k; (**d**)–(**f**): 5 k.

SEM images show that particle size reduction was achieved by both the PLA and PLAL methods (Fig. 5). Size distribution analysis was not possible because of the overlapping and aggregation of the particles.

**Figure 6:**
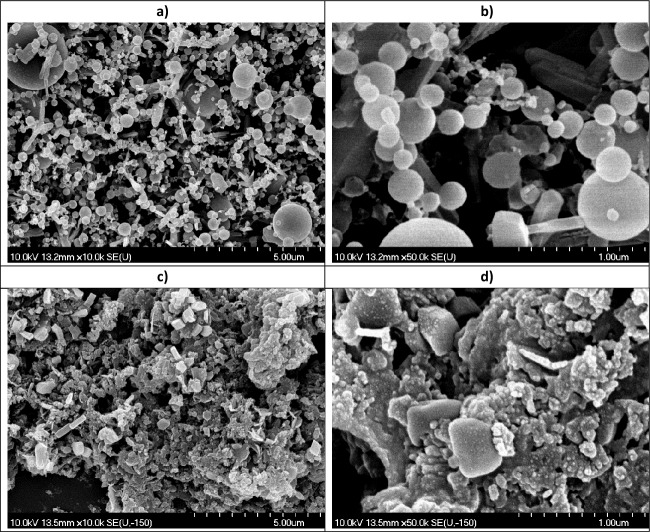
SEM pictures of particles generated by (**a**, **b**) PLA and (**c**, **d**) PLAL. Magnification: (**a**, **c**): 10 k; (**b**, **d**): 50 k.

SEM studies revealed the effect of the medium on the particle morphology. On the one hand, the PLA generated particles were of two types: smooth spheres with a wide range of diameters (~ 0.1–3.0 µm) and pin-like rods (~ 1.0 µm × 0.25 µm × 0.25 µm) (Fig. 6 a,b).

On the other hand, the particles produced by PLAL were typically clumped together in piles, perhaps as a result of drying and some recrystallization. In these particle clusters, there were crystal-like pieces with smooth surfaces and sharp edges, as well as rounded parts and amorphous-like shapeless mass (Fig. 6c,d). Overall, the aggregated particles showed a large surface area.

**Figure 7:**
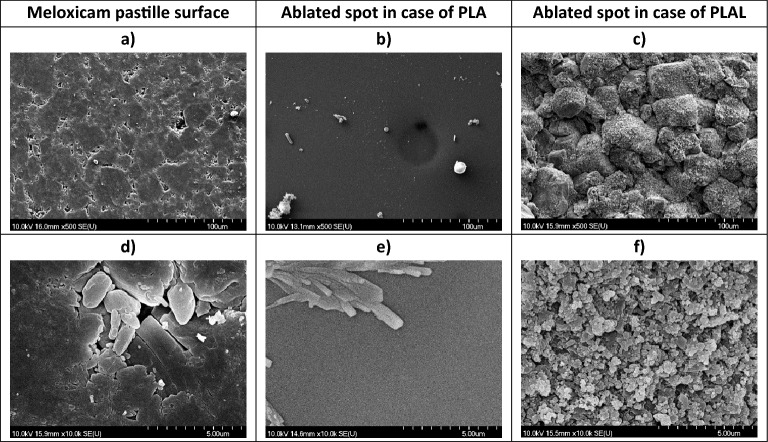
SEM images of (**a**, **d**) the pressed pastille surface (mx. ref.) and the ablation spot after (**b**, **e**) PLA and (**c**, **f**) PLAL. Magnification: (**a**)–(**c**): 500; (**d**)–(**f**): 10 k.

We also compared the ablated surfaces with the untouched surface of the pressed meloxicam pastille (Fig. 7a,d). After ablation in air (PLA), the irradiated spot was smooth with a few scattered particles (Fig. 7b,e). After ablation in distilled water (PLAL), the irradiated spot displayed the compressed original meloxicam crystals, covered with sub-micrometre-size fine structure on the surface (Fig. 7c,f).

**Figure 8:**
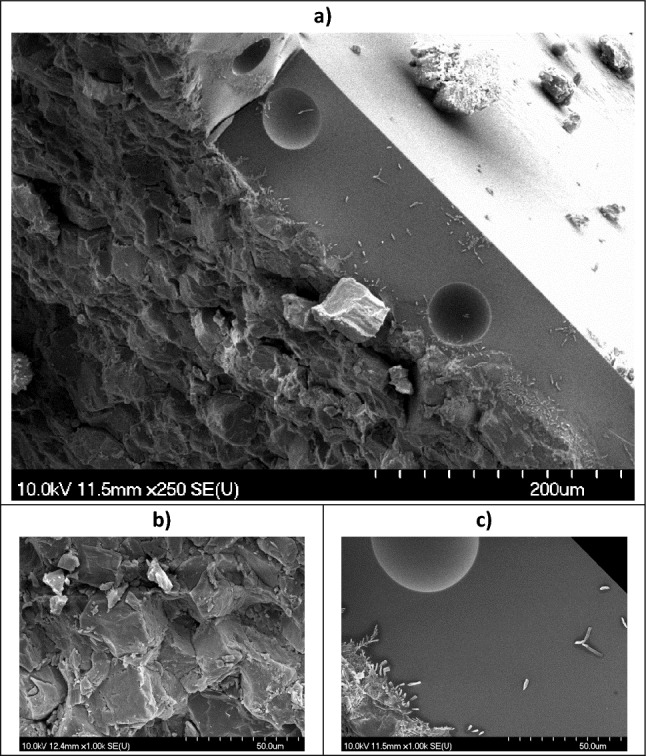
(**a**–**c**) SEM images of the cross-section of the ablation spot after PLA: (**b**) bulk part of the pastille and (**c**) dense, heat affected zone. Magnification: (**a**): 250; (**b**, **c**): 1 k.

By inspecting the cross-section of the ablated pastille after PLA (Fig. 8a), we can distinguish two parts. At the top, there is a well-defined, dense, ~ 100 µm thick layer, with a few hemispherical cavities (Fig. 8c). This resembles a heat affected zone (HAZ), where melting and fast resolidification took place. Under this, the bulk part of the pastille consists of coarse particles of the compacted powder (Fig. 8b).

**Figure 9:**
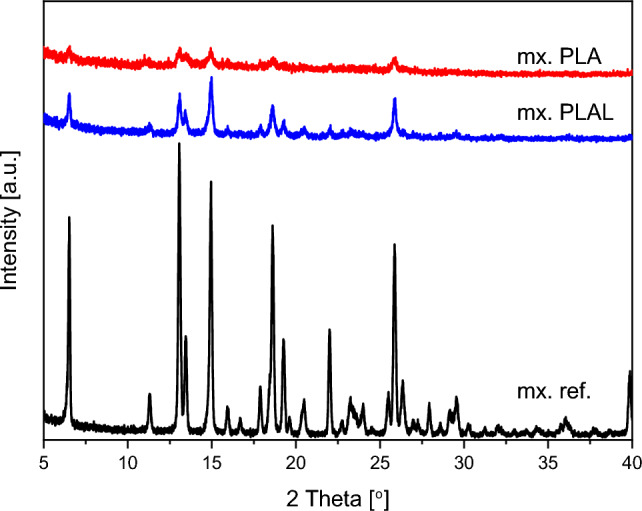
XRPD pattern of PLA (mx. PLA) and PLAL (mx. PLAL) generated particles and the original meloxicam (mx. ref.) powder as reference (for equal quantities of material).

The crystallinity of the particles generated by PLA and PLAL were studied by X-ray diffraction spectroscopy (Fig. 9). In all cases, equal amounts of dry samples and reference were tested. In both the PLA and PLAL samples, the strong, characteristic peaks were present at the expected angles, while the weaker peaks got lost in the noise. Generally, the intensity of the XRPD peaks for the laser ablated particles was significantly reduced compared to the reference. For example, the intensity of the peak at ∼25.94°was reduced to its 1/4th and 1/10th for PLAL and PLA samples, respectively. No noticeable broadening or diffuse shallowing of the peaks was observed.

**Figure 10:**
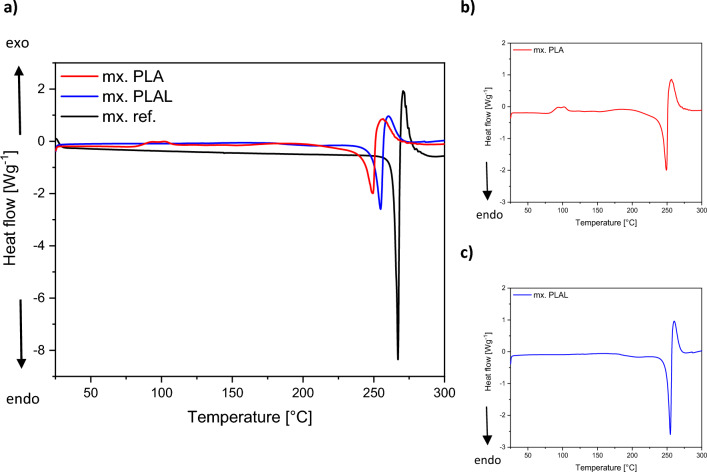
(**a**) DSC curves of PLA (mx. PLA) and PLAL (mx. PLAL) generated particles and the original meloxicam (mx. ref.) powder as reference presented together for comparability. The DSC curves of (**b**) PLA and (**c**) PLAL presented separately for improved visibility.

In the DSC curves of the laser ablated samples (Fig. 10a), the endotherm melting peaks shifted towards lower temperatures, and also flattened and broadened, indicating a decrease in crystallinity. The 15.78 °C lower T_m_ of the PLA generated particles compared to the reference clearly indicates amorphization. The slightly smaller 10.31 °C decrease in T_m_ for the PLAL particles also implies amorphization, even if to a lesser extent. Compared to the PLAL generated particles, the more significant amorphization found for the PLA generated particles using DSC is consistent with the more pronounced crystallinity loss observed in the XRPD for these particles. Numerically, the endothermic peaks for PLA, PLAL and the reference samples were at 248.91 °C, 254.47 °C and 264.78 °C, respectively. Moreover, the PLA curve showed a low-intensity exothermic double peak at around 90–100 °C (Fig. 10b), which could characterize some sort of crystallization processes. In addition, for PLAL there was a saddle point around ∼ 180 °C (Fig. 10c); this type of change is typically associated with glass transitions^53,54^.

## Discussion

Based on the investigation of the chemical composition we conclude that PLA introduced minimal degradation in the final product, while by PLAL a significant amount of meloxicam impurity B was produced. The increased degradation can be the result of laser fragmentation, which occurs when the ablation generated particles are dispersed in the media and they interact with the incoming pulses. The pulsed laser irradiation induced decomposition of powders dispersed in liquids is a well-known and thoroughly studied phenomenon^34–37^. In our case, laser fragmentation could be a secondary particle size reduction effect, due to the high frequency of the laser pulses and the lack of constant particle removal. The FTIR and Raman spectra of the PLA/PLAL generated particles mostly matched the reference, indicating the predominant presence of meloxicam in the ablated samples. Our measurements also demonstrated the limits of FTIR and Raman spectroscopies, which could not detect the presence of impurity being part of the original molecule. Meloxicam impurity B in the ablated samples could only be identified by HPLC–MS. The relative intensities of the FTIR peaks were slightly different from the reference for the PLA sample, but this could not indicate the presence of degradation products, as this was not observed at all in the spectra of the PLAL sample, which contained more degradation products according to the HPLC–MS measurements. Apart from chemical changes, changes in crystallinity or structure can also alter the FTIR spectra, therefore we suspect that the FTIR spectra of the PLA samples also indicate this^47,55–57^.

Particle size reduction was achieved by both the PLA and the PLAL method: mainly sub-micrometre sized particles were generated. In case of PLAL, aggregation took place, possibly during the drying process.

**Figure 11:**
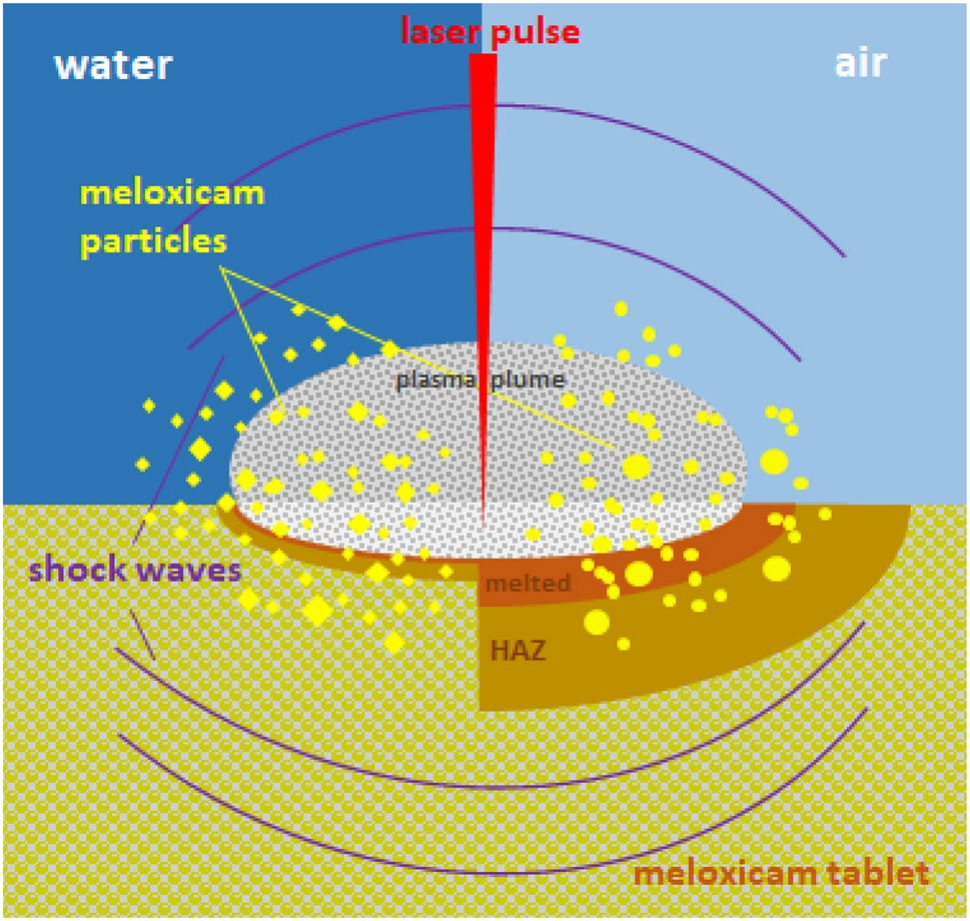
Schematics of the PLAL and PLA ablation mechanisms of meloxicam tablet.

The formation of particles with the variety of shapes can be best explained by the effect of the shock wave induced during laser ablation^44^. When the laser pulse interacts with the target material, a small amount of material is rapidly heated to a high temperature, causing it to vaporize and form a plasma. The plasma expands quickly, generating a recoil pressure and a shock wave that propagates through the target material. The recoil forces acting at a distance from the hottest region can flake off particles with retained chemical integrity, and this is how we could get meloxicam particles. However, the different morphologies of the PLA and PLAL particles indicate that mechanical and thermal effects contribute differently to the ablation mechanisms in air and liquid. Presumably, due to the different thermal conductivity of the two media, in air, thermal effects are considerable, while in liquids, mechanical fracturing is the main process during ablation. In water environment the confinement of the plasma results in a higher plasma temperature, and the cavitation bubbles may oscillate a few times for a couple of hundred microseconds. We assume that the water may cool down the area close to the irradiated zone or even the ablated zone before the next laser pulse arrives. In air the multiple irradiations will result in a higher temperature and probably also a deeper heat-affected zone leading to the ejection of molten material (Fig. 11.).

Particles produced by PLA were mostly smooth spheres with a wide particle size distribution. The spherical shape, which is an energetically favourable shape due to its minimal surface area, could be the result of the rapid resolidification of the molten droplet. The appearance of the ablation spot also indicates the influence of thermal effects. The SEM images of ablated pastilles show a molten, dense, and homogenous top layer, clearly separated from the underlying crystalline bulk. Although meloxicam decomposes at its melting point^41^, it is possible that during the femtosecond laser ablation, the pressure and temperature parameters fall in a special range that enables the phase change without degradation. This assumption is in good agreement with our observation that only minor degradation was detected with HPLC–MS. We assume that in the phase diagram of meloxicam there is a certain pressure range where melting can occur below the decomposition temperature.

The particles obtained from the suspension, produced by PLAL, contained crystal-like pieces with smooth surfaces and sharp edges, as well as rounded parts and amorphous-like shapeless mass. However, due to the high frequency of the laser pulses, laser fragmentation is also a mechanism to be considered, as it may play a role in the further size reduction and possible degradation of the already ablated and dispersed particles. Our observations for PLAL, can partially be explained by the photoacoustic damage mechanism caused by tensile stress waves when the absorbing structures are irradiated with a laser pulse under the conditions of stress confinement^58^. Photoacoustic fracture can occur at fluences below the evaporation threshold both in some target regions and in the dispersed particles resulting in spallation of particles without thermal damage^59^.

The rounded edges and the amorphous-like mass could be the sign of some thermal effects.

The XRPD and DSC (shift of the melting peaks to lower temperatures)^46,60–62^ measurements also indicated that the samples produced by laser ablation may contain an amorphous phase in addition to the crystalline phase. The PLA produced particles differed more from the original, but the PLAL generated particles also showed similar trends. It is worth mentioning that beside amorphization, a reduction in crystallite size can also affect the spectra and partially account for increased noise and the broadening or disappearance of peaks^63,64^.

Upon investigating the FTIR, Raman and XRPD spectra, we can conclude that the laser ablated samples kept the polymorphic state of the original meloxicam, which was the enolic form or form I^65^.

## Conclusions

Femtosecond pulsed laser ablation proved to be not only a particle size reduction technique both in distilled water (PLAL) and in air (PLA) but also a unique amorphization technique for pharmaceutical compounds.

The FTIR and Raman spectroscopy studies implied that most of the generated particles were meloxicam. The HPLC–MS investigations revealed the presence of a small (< 5%) amount of meloxicam impurity B in the PLA, and a more significant (∼ 20%) amount in the PLAL generated particles. Regarding the yield we have multiple factors causing uncertainty, but for reference, a rough estimation for obtained particle yield was around 0.2–0.3 mg/s for PLA and 0.2–0.3 mg/min for PLAL, which correspond to 720–1080 mg/h and 12–18 mg/h of product for PLA and PLAL, respectively. However, these data carry large errors and could be improved and optimized for continuous large-scale production. Particle morphology varied with the applied media. On the one hand, smooth spherical particles with a wide particle size distribution were obtained by PLA. On the other hand, aggregated clusters of crystal-like pieces, rounded, amorphous-like particles and shapeless heaps were seen in the sample produced by PLAL. The morphology of the particles and the ablated surfaces suggested that during PLA, melting occurred, and thermal effects accompanied the ablation process, whereas during PLAL, the mechanical effect of the laser-induced shock wave interacting with the surface caused fracturing and thermal effects played only a minor role. In the absence of any reference in the literature for pure amorphous meloxicam, we cannot say for sure, but the XRPD and DSC data indicated the formation of an amorphous meloxicam phase in both media, but it was more pronounced in air. We demonstrated that with femtosecond PLA meloxicam can be melted without the formation of residual degradation products. Based on the FTIR and Raman spectra, we concluded that femtosecond laser ablation did not alter the polymorphic state of the original meloxicam, the enolic form was preserved. The meloxicam particles produced by femtosecond PLA could be ideal for excipient-free pulmonary administration due to the aerodynamically favourable shape and size range. Alternatively, the PLAL generated meloxicam particles with reduced size could be optimal for the preparation of a predispersion, which can then be further developed for different administration routes (e.g. *per os*, pulmonary or nasal) by adding excipients or by other formulation methods. In the future, the PLAL setup could be improved, e.g. by continuously removing the generated particles with a liquid flow or reducing the frequency and thereby probably avoiding degradation.

## Data Availability

The datasets generated and/or analyzed during the current study are available from the corresponding author on reasonable request.
